# Risk factors associated with diabetic retinopathy in patients with diabetes mellitus type 2

**DOI:** 10.1186/1756-0500-3-153

**Published:** 2010-06-01

**Authors:** Irini P Chatziralli, Theodoros N Sergentanis, Petros Keryttopoulos, Nikolaos Vatkalis, Antonis Agorastos, Leonidas Papazisis

**Affiliations:** 1 Department of Ophthalmology, Veroia General Hospital, Veroia, Greece; 2 School of Medicine, National University of Athens, Greece; 3 Department of Internal Medicine, Veroia General Hospital, Veroia, Greece

## Abstract

**Background:**

Diabetes mellitus (DM) is associated with microvascular complications, such as diabetic retinopathy (DR). DR is one of the main causes of visual loss in individuals aged 20-64 years old. This study aims to investigate the independent associations between the stage of DR and a variety of possible risk factors, including years since DM diagnosis, HbA_1c _levels, the coexistence of hypertension, age and gender.

**Findings:**

120 patients were recruited in the Department of Internal Medicine, Veroia General Hospital, Veroia, Greece, and the DR stage was defined by an ophthalmologist. Afterwards, the DR association with the aforementioned factors was examined. Univariate and multivariate analysis (multivariate ordinal logistic regression) was performed. At the univariate analysis, there was a positive association between DR severity and age (Spearman's rho = 0.4869, p < 0.0001), years since DM diagnosis (Spearman's rho = 0.6877, p < 0.0001), HbA_1c _levels (Spearman's rho = 0.6315, p < 0.0001), history of hypertension (2.47 ± 1.37 vs. 0.50 ± 0.80 for patients without hypertension; p < 0.0001) and male sex (2.56 ± 1.41 vs. 2.05 ± 1.45 for female patients; p = 0.045, MWW). All these factors, except for age, retained their statistical significance at the multivariate ordinal logistic model.

**Conclusions:**

Years since DM diagnosis, hypertension, HbA_1c _levels and male sex are independently associated with severe DR. The effect of age seems to reflect a confounding association.

## Background

Diabetes mellitus (DM) is associated with microvascular complications, such as diabetic retinopathy (DR). DR is one of the main causes of visual loss in individuals aged 20-64 years old [1] and is present in more than 77% of patients with DM type 2 who survive for over 20 years with the disease [2]. Many factors have been associated with the progression and severity of DR, such as DM duration [3,4], the control of serum glucose levels [4-6], hypertension [4,7] or gender [4]. So far the most solid evidence concerning the risk of DR pertains to chronic hyperglycemia; interestingly, in insulin users with DM type 2 (i.e., more severe cases), DR occurs in approximately 84.5% of patients after 15 years of the disease [6,8]. In this study, we aim to investigate the independent associations between the stage of DR and a variety of risk factors, including years since DM diagnosis, HbA_1c _levels, the coexistence of hypertension, age and gender.

## Materials and methods

120 consecutive patients with type 2 DM (56 men and 64 women) participated in the study (age range: 42-89 years old, median: 73.5 years). All consecutive patients were recruited (during their routine follow-up) in the Department of Internal Medicine, Veroia General Hospital, Veroia, Greece, during the time period April 2009 to September 2009. A person was considered as known diabetic, if there was an informational letter from a diabetologist. All participants underwent a comprehensive dilated fundus examination to detect DR by indirect ophthalmoscopy. DR was clinically graded in accordance with the International Clinical Diabetic Retinopathy guidelines [9]. The examined risk factors included demographic and clinical parameters. The demographic risk factors studied were age and gender. The clinical factors were the following: years since diagnosis of DM, HbA_1c _levels and coexistence of hypertension.

The severity of DR was treated as an ordinal variable. Specifically, 0: without findings, 1: mild non-proliferative DR, 2: moderate non-priliferative DR, 3: severe non-proliferative DR, 4: proliferative DR. Similarly, the variable concerning years since diagnosis of DM was treated as an ordinal variable (0: 0-5 years, 1: 5-10 years, 2: 10-15 years, 3: 15-20 years, 4: more than 20 years). Accordingly, HbA_1c _levels were treated as an ordinal variable (0: <7%, 1: 7-8.5%, 2: >8.5%).

The statistical analysis encompassed two steps: univariate and multivariate analysis. At the univariate analysis, the associations between the severity of DR and the study variables (age, gender, HbA_1c _levels, years since diagnosis of DM and hypertension) were appropriately evaluated through non-parametric statistics (Mann-Whitney-Wilcoxon test for independent samples, designated as MWW for reasons of brevity, or Spearman's rank correlation coefficient).

At the multivariate analysis, multivariate ordinal logistic regression was performed. The severity of DR was treated as the dependent variable. The variables which were proven significant at the univariate analysis were entered as independent variables in the model; backward selection of variables was performed so as to reach the most parsimonious model. The satisfaction of the proportionality-of-odds assumption was assessed with the appropriate likelihood ratio test. Statistical analysis was performed with STATA 8.0 statistical software (Stata Corporation, College Station, TX, USA).

This study is in accordance with the Declaration of Helsinki and has been approved by the local Ethics Committee. Written informed consent was obtained from all patients. The authors declare no conflict of interest.

## Results

**Figure 1:**
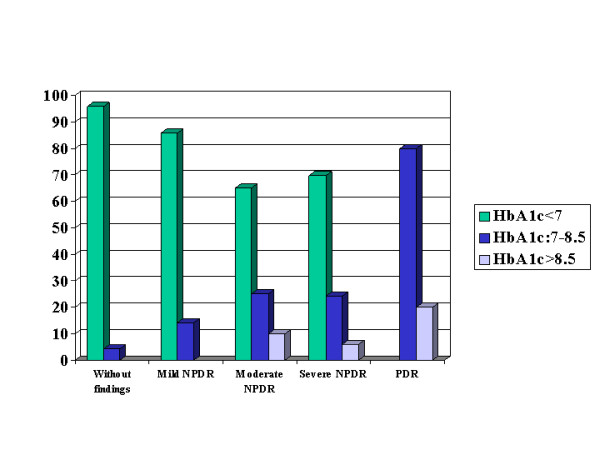
**Graph presenting the positive association between the severity of DR and HbA_1c _levels**.

At the univariate analysis, the severity of DR was positively associated with patients' age (Spearman's rho = 0.4869, p < 0.0001), years since DM diagnosis (Spearman's rho = 0.6877, p < 0.0001, Table 1), HbA_1c _levels (Spearman's rho = 0.6315, p < 0.0001, Figure 1). Male patients exhibited more advanced DR (2.56 ± 1.41 vs. 2.05 ± 1.45 for female patients; p = 0.045, MWW). Patients with coexisting hypertension presented with more severe DR (2.47 ± 1.37 vs. 0.50 ± 0.80 for patients without hypertension; p < 0.0001, MWW).

**Table 1:** Detailed results on the association between the severity of DR and years since diagnosis of DM.

	Duration of DM				
	
Diabetic Retinopathy stage	0-5 years	5-10 years	10-15 years	15-20 years	>20 years
	
	n = 18	n = 10	n = 18	n = 26	n = 48
Without findings	88.9% (16/18)	40% (4/10)	16.7% (3/18)		

Mild Non-Proliferative DR	11.1% (2/18)	40% (4/10)	27.8% (5/18)		6.3% (3/48)

Moderate Non-proliferative DR		20% (2/10)	22.2% (4/18)	11.5% (3/26)	22.9% (11/48)

Severe Non-proliferative DR			22.2% (4/18)	65.4% (17/26)	25.0% (12/48)

Proliferative DR			11.1% (2/18)	23.1% (6/26)	45.8% (22/48)

The results of the multivariate ordinal logistic regression are presented in Table 2. All variables, except for age, retained their statistically significant associations with the severity of DR at the multivariate approach.

**Table 2:** Results of the multivariate ordinal logistic regression

Variables	Category or increment	OR (95% CI)	p-value
Gender	Male vs. Female	3.57 (1.67-7.62)	0.001
Hypertension	Yes vs. No	4.49 (1.15-17.49)	0.030
Years since diagnosis of DM	1 level increase	3.37 (2.34-4.85)	<0.001
HbA_1c_	1 level increase	4.53 (2.11-9.72)	<0.001

## Discussion

The duration of DM appears as a meaningful predictor for DR and its severity [3,4,10] however, cases free of DR are occasionally reported despite long duration of DM [8]. Carefully examining the descriptive statistics of our study sample, it is astonishing that 45.8% of patients who were diagnosed with DM twenty or more years ago exhibit proliferative DR; on the contrary, 88.9% of patients who have been diagnosed with DM since 0-5 years are free of DR.

The role of chronic hyperglycemia in the development of DR has also been well established [4-6]. High HbA_1c _levels are closely associated with severe DR in our study. Decrease in HbA_1c _concentrations by 1% leads to an estimated reduction of 30% in the risk of microvascular complications [8].

Hypertension is an important risk factor for the onset and progression of the disease, and in most studies it is an independent risk factor for DR [4,6,7,10]. The UK Prospective Diabetes Study (UKPDS) demonstrated that blood pressure control is associated with a reduction in DR incidence; the relative risk for DR is 1.5 concerning systolic pressure between 125-139 mmHg and 2.8 for systolic pressure higher than 140 mm Hg [6].

In our study, male patients exhibited more advanced DR; this finding seems distinct than that reported by previous studies, according to which male gender was related with the presence of DR, but not its severity [4,10].

Concerning the role of age, it is worth commenting on our study findings. Age was associated with the severity of DR at the univariate analysis but lost its significance at the multivariate model. Most probably, this reflects a confounding effect of age, as older age and longer duration of DM are two factors closely associated with each other. As a result, age does not seem to represent an independent risk factor for severe DR.

A limitation of the present study was that DR grading was based on fundoscopy and not on fundus photography grading. This could have resulted in underestimation of the prevalence and severity of DR. In addition, a considerable limitation of this study pertains to the relatively small sample size; nevertheless, the fact that the results persisted at the multivariate approach points to the validity of the findings presented herein.

## Conclusions

In conclusion, this study demonstrates that male gender, coexistence of hypertension, years since diagnosis of DM and HbA_1c _represent independent risk factors for severe DR.

## List of abbreviations

DM: Diabetes Mellitus; DR: Diabetic Retinopathy; MWW: Mann-Whitney-Wilcoxon; UKPDS: UK Prospective Diabetes Study.

## Competing interests

The authors declare that they have no competing interests.

## Authors' contributions

IPC conceived the idea of the study, designed the study, performed fundoscopy and drafted the manuscript. TNS performed the statistical analysis and drafted the manuscript. PK participated in the design of the study and drafted the manuscript. NV participated in the recruitment of patients and revised the manuscript critically for important intellectual content. AA participated in data collection and revised the manuscript critically for important intellectual content. LP participated in the design of the study and its coordination, helped to draft the manuscript and has given final approval of the version to be published. All authors read and approved the final manuscript.
